# The intersectional jeopardy of disability, gender and sexual and reproductive health: experiences and recommendations of women and men with disabilities in Northern Uganda

**DOI:** 10.1080/26410397.2020.1772654

**Published:** 2020-06-19

**Authors:** Muriel Mac-Seing, Kate Zinszer, Bryan Eryong, Emma Ajok, Olivier Ferlatte, Christina Zarowsky

**Affiliations:** a PhD Candidate, Department of Social and Preventive Medicine, School of Public Health, Université de Montréal, Montreal, Canada; Centre de recherche en santé publique, Université de Montréal et CIUSSS du Centre-Sud-de-l’Île-de-Montréal, Montreal, Canada.; b Associate Professor, Department of Social and Preventive Medicine, School of PublicHealth, Université de Montréal, Montreal, Canada; Centre de recherche en santé publique, Université de Montréal et CIUSSS du Centre-Sud-de-l’Île-de-Montréal, Montreal, Canada; c Independent Researcher, Gulu, Uganda; d Associate Professor, Department of Social and Preventive Medicine, School of Public Health, Université de Montréal, Montreal, Canada; Centre de recherche en santé publique, Université de Montréal et CIUSSS du Centre-Sud-de-l’Île-de-Montréal, Montreal, Canada; e Full Professor, Department of Social and Preventive Medicine, School of Public Health, Université de Montréal, Montreal, Canada; Centre de recherche en santé publique, Université de Montréal et CIUSSS du Centre-Sud-de-l’Île-de-Montréal, Montreal, Canada; University of the Western Cape, Bellville, South Africa

**Keywords:** intersectionality, disability, gender, sexual and reproductive health and rights, health equity, Uganda

## Abstract

The 2030 Sustainable Development Goals committed to “Leave No One Behind” regardless of social identity. While access to sexual and reproductive health (SRH) services has improved globally, people with disabilities continue to face enormous barriers to SRH, infringing on their SRH rights (SRHR). Uganda adopted pro-disability legislation to promote the rights of people with disabilities. Despite these legal instruments, SRHR of people with disabilities continue to be violated and denied. To address this, we sought to understand and document how people with disabilities perceive the relationships between their use of SRH services, legislation, and health policy in three districts of the post-conflict Northern region of Uganda. Through an intersectionality-informed analysis, we interviewed 32 women and men with different types of impairments (physical, sensory and mental) and conducted two focus groups with 12 hearing and non-hearing disabled people as well as non-participant observations at seven health facilities. We found that disabled people’s access to SHR services is shaped by the intersections of gender, disability, and violence, and that individuals with disabilities experienced discrimination across both private-not-for-profit and public health facilities. They also encountered numerous physical, attitudinal, and communication accessibility barriers. Despite policy implementation challenges, people with disabilities expected to exercise their rights and made concrete multi-level recommendations to redress situations of inequity and disadvantages in SRH service utilisation. Intersectionality revealed blind spots in policy implementation and service utilisation gaps. Universal health coverage can be operationalised in actionable measures where its universality meets with social justice.

## Résumé

Les objectifs de développement durable 2030 prévoient de « ne laisser personne de côté », sans distinction d'identité sociale. Si l'accès aux services de santé sexuelle et reproductive (SSR) s'est élargi dans le monde, les personnes handicapées rencontrent encore d'énormes obstacles pour obtenir des soins dans ce domaine, ce qui viole leurs droits à la SSR. L'Ouganda a adopté une législation qui favorise les droits des personnes handicapées. En dépit de ces instruments juridiques, les droits à la SSR des personnes handicapées continuent d'être enfreints et déniés. Pour remédier à ce problème, nous avons cherché à comprendre et à documenter comment les personnes handicapées perçoivent les liens entre leur utilisation des services de SSR, la législation et la politique de santé dans trois districts en contexte de post-conflit au nord de l'Ouganda. Par le biais d'une analyse intersectionnelle, nous avons interviewé 32 femmes et hommes avec différents types de handicaps (physiques, sensoriels et mentaux) et conduit deux groupes de discussion avec 12 personnes handicapées entendantes et malentendantes ainsi que des observations non-participantes de sept structures de santé. Nous avons constaté que l'accès des personnes handicapées aux services de SSR est modelé par les intersections du genre, du handicap et de la violence, et que les individus handicapés se heurtent à une discrimination aussi bien dans les structures sanitaires publics que privés à but non lucratif. Ils rencontrent aussi de nombreux obstacles à l'accessibilité, de nature physique ou en lien avec les attitudes et la communication. Malgré des difficultés dans l'application des politiques, les personnes handicapées s'attendaient à exercer leurs droits et ont formulé des recommandations concrètes sur plusieurs niveaux afin de corriger les situations d'iniquité et de désavantage dans l'utilisation des services de SSR. L'intersectionnalité a révélé des angles morts dans la mise en œuvre des politiques et des lacunes dans le recours aux services. La couverture de santé universelle peut être atteinte avec la mise en place de mesures réalisables où son universalité sert la justice sociale.

## **Resumen**

Los Objetivos de Desarrollo Sostenible 2030 se comprometieron a “No dejar a nadie atrás”, independientemente de su identidad social. Aunque el acceso a los servicios de salud sexual y reproductiva (SSR) ha mejorado mundialmente, las personas con discapacidad continúan enfrentando enormes barreras a la SSR, que infringen en sus derechos de SSR (SDSR). Uganda adoptó legislación a favor de las personas con discapacidad para promover sus derechos. A pesar de estos instrumentos legislativos, SDSR de personas con discapacidad continúan siendo violados y negados. Para abordar ese problema, buscamos entender y documentar cómo las personas con discapacidad perciben las relaciones entre su uso de servicios de SSR, la legislación y la política sobre salud en tres distritos de la región septentrional de Uganda postconflicto. Por medio de un análisis informado por interseccionalidad, entrevistamos a 32 mujeres y hombres con diferentes tipos de discapacidad (física, sensorial y mental) y realizamos dos discusiones en grupos focales con 12 personas discapacitadas oyentes y sordas, así como observaciones no participantes en siete unidades de salud. Encontramos que el acceso de las personas discapacitadas a los servicios de SSR es afectado por las intersecciones de género, discapacidad y violencia, y que las personas con discapacidad sufrieron discriminación tanto en unidades de salud del sector privado sin fines de lucro como en las del sector público. Además, enfrentaron numerosas barreras físicas, actitudinales y de comunicación a la accesibilidad. A pesar de los retos de aplicar las políticas, las personas con discapacidad esperaban poder ejercer sus derechos e hicieron recomendaciones concretas en múltiples niveles para rectificar situaciones de inequidad y desventajas en el uso de servicios de SSR. La interseccionalidad reveló puntos ciegos en la aplicación de políticas y brechas en el uso de servicios. Es posible operacionalizar la cobertura médica universal con medidas viables cuando su universalidad coincide con la justicia social.

## Introduction

Access to sexual and reproductive health (SRH) services has improved globally; however, millions continue to have unmet SRH needs, particularly those living in poverty and rural areas, including people with disabilities.^1^ The sexual and reproductive health and rights (SRHR) of people with disabilities remain violated and silenced.^2^ Approximately one billion people worldwide live with some form of disability (physical, sensory, intellectual or mental) with 80% of disabled individuals living in low- and middle-income countries.^3^ The 2030 Sustainable Development Goals (SDGs), adopted in 2015 by the international community, underscore the need to “leave no one behind”, regardless of gender, age, ability, wealth, or geographic location.^4^ Among these goals, at the intersection of SDG5, focusing on gender equality and the empowerment of women and girls, SDG3 promotes healthy lives, including SRH which is intertwined with and contributes to the attainment of universal health coverage. (UHC)^1^ When SRHR are examined from a disability and gender lens, pervasive SRHR violations have been reported to severely affect women and girls with disabilities, ranging from forced and/or coerced sterilisation, gender-based violence to lack of access to basic SRH services and information.^5^

Twenty-five years after the recognition of SRHR at the First International Conference on Population and Development (ICPD) in Cairo, many governments, researchers, activists, coalitions of marginalised groups, and development partners reconvened in Nairobi in 2019. They reviewed ICPD progress related to SRHR and shared positive outcomes: global maternal mortality and HIV prevalence have both decreased, while access to family planning has increased.^6^ However, gender-based violence continues unabated and still disproportionally affects adolescents and young women, especially women who live in conflict and war zones, as well as girls and young women with disabilities who “experienc[e] four times more violence than those without disabilities”.^6^ Compared to non-disabled people, women and men with disabilities experience multiple physical, attitudinal, and structural barriers infringing their SRHR, globally.^3^ They encounter additional obstacles to accessing maternal and reproductive health services,^7,8^ and are at increased risk of HIV^9^ and of multiple forms of violence. People with intellectual disabilities and people living with mental health illnesses are particularly at risk of violence.^10^ In sub-Saharan Africa, people with disabilities have been reported to encounter all the above barriers, combined with poor access to basic SRH services and health system infrastructures.^7,8,11,12^

After years of debate among the United Nations Member States about how to promote and protect the rights of people with disabilities, the Convention on the Rights of Persons with Disabilities (CRPD) was adopted in 2006 and entered into force in 2008.^13^ People with disabilities are referred to as “people who have long-term physical, mental, intellectual, or sensory impairments which in interaction with various barriers may hinder their full and effective participation in society on an equal basis with others”.^14^ Legally, the CRPD seeks to compensate the historical disadvantages experienced by people with disabilities by providing guiding principles, such as non-discrimination and specific articles on rights, for example social participation, health, education, and employment.^13^ To date, more than 180 Member States have ratified the CRPD,^15^ including Uganda, which recently emerged from a 20-year armed conflict. The conflict largely affected the Northern region. The health system was severely weakened, health programmes had to be rebuilt, while gender-based violence and unwanted pregnancies were high and access to safe motherhood jeopardised, affecting most women and children.^16,17^ Among sub-Saharan African countries, Uganda is cited as an example of a disability rights promoter.^12,18^ The adoption of its Disability Act in 2006 and the ratification of the CRPD in 2008 are embedded in a legal space that dates from the promulgation of its Constitution in 1995 and its amendment in 2005, which enshrined the rights of people with disabilities.^19^ However, despite these legal tools, the presence of a National Council on Disability^20^ and a representation of disabled elected officials at different governmental levels, concrete actions aimed at protecting the rights of people with disabilities are still lacking.^12^ People with disabilities in Uganda continue to have limited access to disability-appropriate and sensitive SRH services and face high rates of discrimination when accessing services, coupled with structural barriers such as service costs.^7,11^

A comprehensive study of national policies helps better understand the trajectories of these policies and the interactions among agenda-setting, policy formulation, implementation, evaluation, and policy outcomes.^21^ The literature examining public policy and human rights, in the context of health, underscores the crucial role these play in anti-discrimination measures and in the provision of services by the state.^22^ While this is important and necessary, it is insufficient to analyse policy in a linear fashion when these interactions are complex and power structures influence policy and social outcomes. To address social inequities and multiple interconnected discriminations experienced by people with disabilities,^5^ Intersectionality-Based Policy Analysis (IBPA) offers a flexible framework to assist researchers and policy actors in bringing attention to intersecting social identities, diverse knowledges, multi-level factors, and a conscious exploration of complex policy issues for transformative policy solutions, beyond simply describing the problem.^23^ Intersectionality addresses the relationships between intertwined social identities, social inequities, power dynamics, social context, and complexity.^24^ Rooted in a long and deep history of Black, Indigenous and third world feminism as well as queer and postcolonial theory, intersectionality is a framework and research paradigm for understanding differences and resisting essentialisation of differences.^25^ The term was first coined in 1989 to address the multiple discriminations faced by Black American women workers who fell outside of the protection of anti-racism and anti-sexism legislation.^26,27^

The lack of data on the relationships between legislation, health policy and utilisation of SRH services by people with disabilities in sub-Saharan Africa is a major gap in the literature, in particular in post-conflict settings where access to and utilisation of services by affected populations are jeopardised.^28^ Framed within the conceptual and methodological context described above, the study reported here aimed to understand and document how people with disabilities perceive the relationships between their utilisation of SRH services, legislation, and health policy in the post-conflict Northern region of Uganda. We were interested in exploring their awareness of the pro-disability legislation and policy implementation, their perceptions of possible inequities related to SRH service utilisation and their recommendations on how to reduce these inequities. This paper reports the qualitative findings related to the perceptions of people with disabilities from the larger body of evidence of a study using mixed methods, which also involved other study participants, namely health service providers, local disabled people’s organisations, international organisations, and national policy-makers.

## Methods

Our study was conducted in the districts of Gulu, Amuru, and Omoro in the Northern region of Uganda. Through a multiple “instrumental” case study design,^29^ our case was defined as the post-conflict Northern region, and the multiple cases include seven health facilities of two different types, the private-not-for-profit facilities (which are faith-based) and public health facilities, as shown in Table 1. Given the instrumental nature of the case study, the focus of this study was not to examine the intrinsic organisation of health facilities, but rather to use them as an “instrument” to develop a better understanding of the perceptions of people with disabilities when they use SRH services. Field research, conducted from November 2017 to April 2018, consisted of three main phases. Phase 1 aimed at learning more about the local context and identifying key knowledge brokers. Phase 2 was dedicated to community mobilisation, recruitment of study participants and data collection. Phase 3 focused on the dissemination of preliminary findings (see Supplementary File 1 for detailed activities). During the fieldwork, a methodological and reflexive logbook documented daily fieldnotes and methodological decisions as well as challenges and reflections on various emerging issues. Our research process was appraised using the Consolidated Criteria for Reporting Qualitative Research (COREQ), a widely used tool to assess rigour in qualitative research (Supplementary File 2).^30^

**Table 1.** Health facilities included in the case study

	Private-not-for-profit health facilities	Public health facilities
Gulu District	Referral Hospital (1)	Referral Hospital (1)
Amuru District	Health Centre Level III (2)	Health Centre Level III (1)
Omoro District	Health Centre Level III (1)	Health Centre Level III (1)
Total	4	3

### Positioning of researchers

The researcher MMS has worked for several years in sub-Saharan Africa with people with disabilities, advocating for their disability rights and SRHR within different international platforms and alongside disabled people’s organisations. Prior to this study, MMS had not worked in Uganda. BE and EA are both Ugandans and speak English and several local languages. They are young social science undergraduates and have worked as research assistants in qualitative research and with people with disabilities. CZ and KZ are supervising the work of MMS in the context of her mixed methods study. Both have extensive research experience in Uganda and in working with vulnerable populations. OF is a queer scholar who works with populations marginalised because of their sexuality or gender identities and is one of the authors of the IPBA framework.

### Study participants

Because of our commitment to include people with a diversity of experiences, we consciously opted to recruit people with different types of impairments (physical, vision, hearing, mental and intellectual) living in the catchment areas of the seven health facilities. The main selection criteria were for adults with disabilities consenting to participate and answer the research questions on their own, without the presence of, nor recourse to, a third party. Purposive sampling sought maximum variation in disability and districts, while ensuring a gender-balanced sample. Village Health Teams (VHT) and disabled volunteers helped in community mobilisation and the identification of potential study participants. Recruitment of people with disabilities continued until saturation was reached.^31^ A total of 44 individuals with disabilities participated in the study: 32 took part in in-depth semi-structured interviews and 12 participated in two focus groups (one for hearing disabled people (*n* = 6) and one for non-hearing disabled people (*n* = 6)).

### Data collection

We conducted in-depth semi-structured interviews, focus groups, and non-participant observations to triangulate findings.^31^ Data collection tools were first discussed among the core research team members (MMS, BE and EA), and field tested with a focus group of people with disabilities. We developed a glossary of key research and SRH vocabulary for consistency. Interview and focus group guidelines were informed by the IBPA framework and adapted for this research. The guidelines included the two sets of IBPA questions:^23^ (1) descriptive questions related to SRH utilisation by people with disabilities and information on policy implementation processes and (2) transformative questions related to solutions aimed at reducing inequities and promoting social justice (Supplementary files 3-4). All interviews and focus groups were led in English by MMS and translated concurrently by BE and EA into Luo/Acholi. For participants with hearing impairments, a locally qualified Ugandan sign language interpreter, fluent in English and Luo/Acholi, was hired. Each interview and focus group lasted approximately one hour and was audio recorded with the permission of study participants. Both BE and EA were present during the interviews and focus groups and they cross-checked one another’s translations. The following day, they transcribed the translated English parts of the recordings. MMS compared the recordings to the transcriptions for quality assurance. For non-participant observations, health managers were notified prior to this exercise. We spent at least half a day for initial visits in addition to follow-up visits. During our observations, we focused on various aspects of accessibility for each health facility. Daily debriefing sessions were conducted to improve the data collection process.

### Analysis

We adopted a thematic analysis following specific steps.^32^ First, to become familiar with the qualitative dataset, all recordings were listened to, while noting preliminary impressions and thoughts related to data. Based on notes taken, selected recordings were listened to at least twice by MMS. All printed transcriptions were then read and re-read several times, noting additional impressions and initial ideas for codes. Second, using an inductive approach, we performed an initial round of coding to identify and organise data relevant to this research. Third, interview transcripts were imported in QDAMiner 5.0.31 (Provalis) and coding was performed. After all transcripts were coded, we used an iterative inductive-deductive approach, informed by intersectionality, to search for themes. As per the IBPA approach,^23^ when identifying themes, particular attention was paid to how study participants answered the two sets of questions (descriptive and transformative) asked during the in-depth interviews and focus groups. At this stage, connections between codes and broad themes were made. Fourth, MMS reviewed the data to check for the representativeness of themes. Fifth, the themes were reviewed and refined through discussion among the authors. Finally, the results were written up, guided by the IBPA’s key principles^25^: (1) intersecting social identities, (2) multilevel analysis (at micro, meso and macro levels), (3) power structures, (4) time and space (context), (5) diverse knowledges, (6) reflexivity, and (7) social justice and equity. Non-participant observations of health facilities related to accessibility were analysed in relation to emerging themes and compared with the narratives and experiences of study participants when accessing and using SRH services. To disseminate the preliminary findings and to seek feedback from the study’s participants and stakeholders, we hosted five workshop presentations in Northern Uganda.

### Ethical approval

This study received ethical clearance from three nationally approved research ethics committees: the Research Centre at the Hospital Centre of the University of Montreal (17.127-CÉR), the Uganda National Council for Science and Technology (SS-4451), and the Lacor Hospital Institutional and Research Ethics Committee (LHIREC 019/07/2017). All participants provided their consent through the support of a translated written consent form in Luo/Acholi and verbal translation by research assistants. Consent forms and support interview tools were made disability-friendly by using pictogrammes.

## Results

### Demographic data

Of the 32 people with disabilities who were individually interviewed, 53% were women. Eight people out of the 32 disclosed being HIV positive (25%); five out of the eight people living with HIV were women. Thirty-nine percent, 19%, 22% and 22% had physical, vision, hearing and mental/intellectual impairments, respectively. Most had a source of income and were in a relationship. About one third had none to less than six years of formal education, while most had studied for more than six years. Almost all had children. Most had acquired their impairment after birth following illnesses or injuries, except for one person. In the two separate focus groups for hearing and non-hearing people with disabilities, half were women (Table 2).

**Table 2.** Characteristics of participants

Characteristics	In-depth semi-structured interviews *N *= 32 (%)*	Focus groups** N = 2 of 12 people (% based on # people)
**Sex**		
Women	17 (53)	6 (50)
Men	15 (47)	6 (50)
**Impairment**		
Physical	12 (38)	3 (25)
Vision	6 (19)	2 (17)
Hearing	7 (22)	7 (58)
Mental/Intellectual	7 (22)	
**Onset of impairment**		
At birth	1 (3)	
After birth	26 (81)	
Not specified	5 (16)	
**HIV status (self-declared)**		
HIV+	8 (25)	
Women among HIV+	5 (63)	
**District**		
Gulu	10 (31)	
Amuru	15 (47)	
Omoro	7 (22)	
**Marital status**		
Single	6 (19)	
Married/In union	17 (53)	
Separated/Divorced/Widow(er)	9 (28)	
**Education (years)**		
0–3	5 (16)	
04-Jun	6 (19)	
> 6	21 (66)	
**Source of income**		
Yes	27 (84)	
No	5 (16)	
**Having children**		
Yes	27 (84)	
No	5 (16)	

*Rounding might be slightly above or below 100%.

**Only sex and disability data collected.

### Major themes

**Figure 1.**
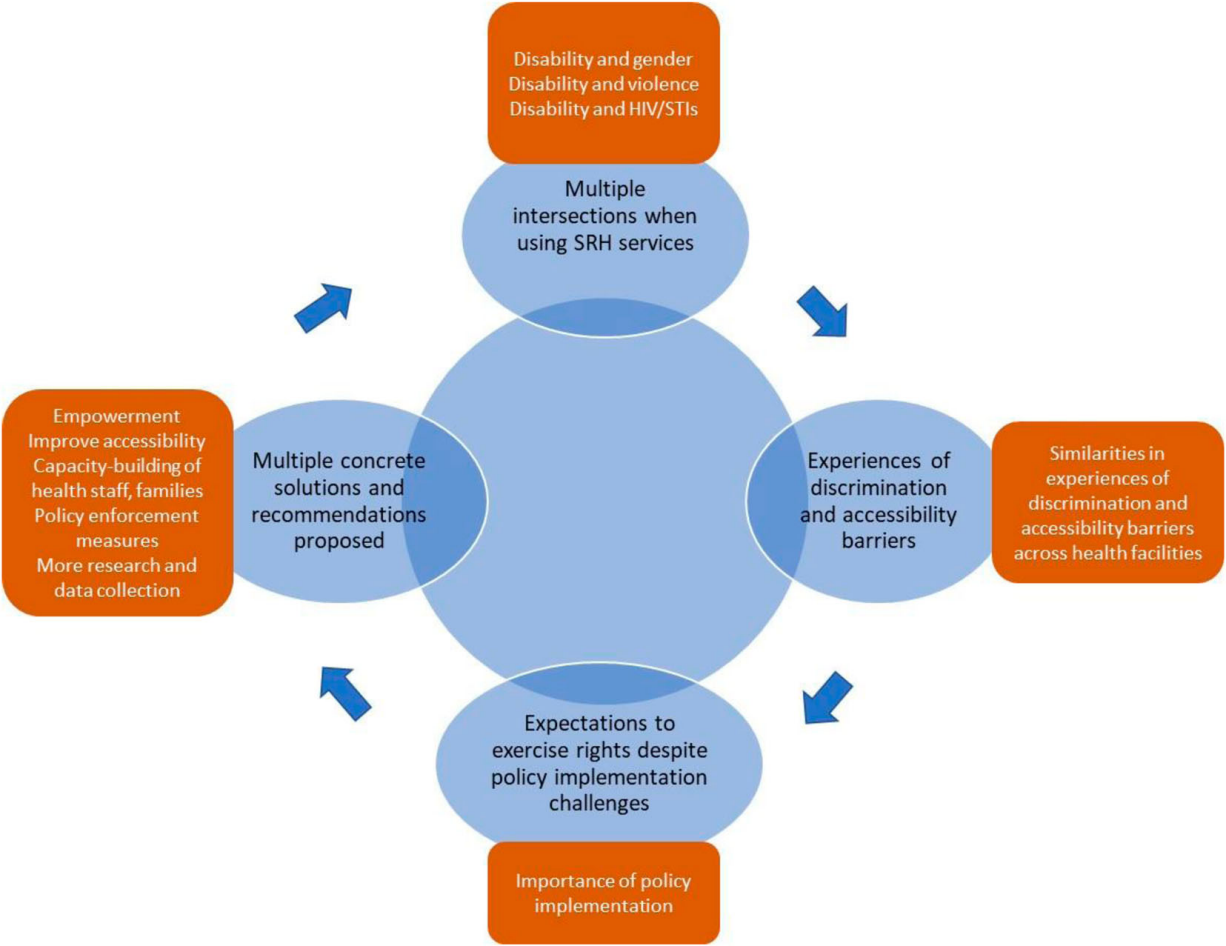
Major themes identified

Embedded in the experiences of women and men with disabilities interviewed, the study identified four interrelated themes (in blue) and sub-themes (in brown) across disability, gender, health facility and district (Figure 1). These main interrelated themes were as follows: (1) multiple intersections when using SRH services; (2) experiences of discrimination and accessibility barriers across health facility type; (3) expectations that people with disabilities exercise their rights despite policy implementation challenges; and (4) multiple concrete recommendations from people with disabilities. Themes and sub-themes are further developed in the following sections.

### Multiple intersections when using SRH services

In discussing their experiences of and perspectives about SRH service utilisation, the connections between the participants’ experiences of disability and other aspects of their identities were evident. People with disabilities, notably women, depicted complex intersections between gender, disability, and experiences of violence, often resulting in unwanted and unexpected health outcomes, such as HIV, sexually transmitted infections (STIs), or pregnancies. Underpinning these simultaneous intersections, the influence of ableism on the lives of women and men with disabilities shaped their struggles to use SRH services, beyond the stigma associated with a disability. While it is difficult to disentangle the various concomitant intersections jeopardising access to and use of SRH services, three main intersections were identified as important to people with disabilities: (1) disability and gender, (2) disability and violence, and (3) disability and HIV/STIs.

#### Disability and gender

When using SRH services, both women and men with disabilities divulged the burden that the service providers’ and societal expectations created as well as of the control this created over their bodies. Health providers and the community discriminated against people with disabilities, constantly challenging their basic sexual rights and capabilities to become pregnant or forge an intimate relationship. As people with disabilities, they were suddenly not expected to fulfil these gendered roles as would any other women and men without disabilities. Study participants felt segregated from mainstream society by being denied their right to use contraception or get married like anyone else.

“In relation to getting a partner, a marriage partner, it is very hard for me as a person with disability to get a woman. People's perceptions are like, when you're disabled, you're not supposed to marry a non-disabled, you are supposed to get a fellow [who is] disabled, and you stay together.” *(Man, physical impairment, Amuru)*

“They [health providers] were saying ‘You who are personally dead, what do you want to space for? Men don’t want you, why do you waste your time, why do you come for family planning, yet you even don’t have men who love you?’. I felt so bad, and then I was wondering, if I go for family planning, does it mean you should be with a man? Am not happy about it … And then, about women with disabilities who go for deliveries in the hospital … . Doctors use the wrong words like ‘These disabled legs are all paralysed, why do you get pregnant?’” *(Focus group, hearing disabled people, Gulu)*

Social norms and the perceptions of inadequacy of people with disabilities were also accompanied by the use of derogatory wording, such as “useless” (Focus group, hearing disabled people, Gulu), “lame” (Man, mental impairment, Gulu), “not normal” (Focus group, non-hearing people, Gulu) and “not fit” (Man, vision impairment, Amuru). These perceptions often led to the assumption that people’s impairment would lead to a complete state of inability to think or perform in society like everyone else and thus, resulting in surprise when this was proven otherwise. A woman and a man with a physical impairment, both from Gulu, reported respectively: “*The nurses and doctors think that when you’re disabled, you’re disabled in your mind, everywhere, in all parts of your body”* and “*Most people thought that maybe the accident has spoilt my manhood. So, when they saw that my wife was pregnant, they were happy to see me going for antenatal [care with her]”*.

In most cases, the social diktat to fit into traditional gender roles was detrimental to women with disabilities. Either they were reduced to their basic gender roles of conventional procreation or they were considered sexual objects. In both cases, women with disabilities were denied the full expression of their sexual and reproductive health rights. In addition, they were considered a burden, coupled with situations of stigmatisation and rejection, furthering their vulnerability through unstable relationships and single motherhood.

“Many women are being left by men. You find that the man can come to me, that he loves me so much, but moment he made me pregnant, he can take off and disappear … People will start saying a lot of words ‘Why did you love that woman with disability, do you think she is going to help you?’ For us who are blind, they will start saying ‘Do you think she can cook for you, she can wash for you, even if she produced [had a child], how is she going to take care of your baby?’ So, when he leaves you, you start struggling with the baby alone.” *(Focus group, hearing disabled people, Gulu)*

### Disability and violence

Compounded with the uneasy experiences based on gender and disability, participants disclosed direct and indirect examples of experiences of violence. Contradicting the belief that people with disabilities constitute a homogenous group, it was demonstrated that people with different types of impairments experienced varying levels of violence and abuse, from being stigmatised and discriminated against to being raped and killed. Participants spoke of the heightened risk of sexual abuse and violence for women with sensory, mental, and intellectual impairments. In some communities, due to their psychotic episodes and most probably combined with a lack of adequate access to mental health care and psychosocial support in the region, women with mental health problems were kept outside of the family home and left to themselves. These situations increased their vulnerability to multiple forms of violence. A woman with mental impairment, from Gulu, said: “*I escaped from my mother, I went away, and I slept somewhere … They had to beat me, I came back naked, there was no clothe on me”.* Other participants shared the followings:

“There are some friends of mine, when we go at the centre … We can be there, and we take soda and there are some guys, some boys who come to them. They forced them to go somewhere and if they go, they abused them sexually … They have sex with them, they only buy them sodas and they don’t even give them any money.” *(Woman, intellectual impairment, Omoro)*

“In villages, you find that sometimes they [women with intellectual/mental impairments] are killed or strangled after being used [raped] … They are separated from the family members. You find the big family is here, and then, you find a disabled woman or man is given a home some distance away from the family members. They become a very good target to these people who are roaming around and who can easily rape them, grab them.” *(Focus group, non-hearing disabled people, Gulu)*

Although a response to address gender-based violence has been progressively put in place in Uganda, including in the Northern region, adapted services for people with disabilities remain limited and are not disability-sensitive.^33^ Non-participant observations revealed that health facilities, which are responsible for delivering the medical part of the response, were poorly accessible to people with various impairments. No disability desk nor signage for people with sensory impairments was available.

### Disability and HIV/STIs

As illustrated above, the intersections between gender, disability, and violence are intricate and could result in health outcomes ranging from STIs to death. In various cases, violence seemed to be the mediating factor in contracting STIs, including HIV. Other factors, such as lack of accessible HIV prevention and services for people with disabilities, may also have contributed to obstructing their full access to and utilisation of HIV-related information and services. Of the five disabled women who declared their HIV status, three lived with physical impairments, one with vision impairment and one with a mental impairment. All women expressed gendered vulnerabilities which further exacerbated their experience of disability intersecting with HIV/STIs and violence.

“The doctors told me to take care of myself. I protect myself, because for us, women with disabilities, most men take advantage of us, they love having sex with us … Because they wait when we're not in good conditions [while experiencing a mental illness crisis], that was when they used the opportunity to abuse us sexually … I was infected with syphilis.” *(Woman, mental impairment, Gulu)*

“When it reaches the time of having sex, he [a man living with HIV] will force on to you, because you don't have energy, he will force on to you … When you realise he has infected you, he will leave you, that is what is happening among women with disabilities.” *(Focus group, hearing disabled people, Gulu)*

## Experiences of discrimination and accessibility barriers

### Similarities in experiences of discrimination and accessibility barriers across health facilities

We observed two types of health facilities where people with disabilities sought SRH services: public health and private-not-for-profit facilities, with the latter mostly supported by faith-based organisations. Both types of health facility provided similar healthcare service packages, ranging from maternal health to more specialised care at the level of referral hospitals. According to study participants, what distinguished both types of health facility in service provision were modern contraception and mental health care services, which were provided by public health facilities but not by private-not-for-profit facilities. Participants also reported that, although health services were supposed to be free and available at all times in public health facilities, health staff were often absent, drugs were out of stock, and patients were referred to external clinics to get their medicine: *“At times, the medicine is not there … You have to go and buy it from outside.”* (Man, physical impairment, Omoro).

Contrary to common assumptions that private and faith-based services are of higher quality,^34^ we found that participants encountered similar obstacles in using SRH services, regardless of the type of health facility. Both private-not-for-profit and public health facilities showed unfriendliness toward users with disabilities, coupled with ableist and demeaning comments. Study participants also described being stigmatised as a result of physical (e.g. lack of assistive devices) and communication (e.g. sign language interpretation) barriers. Women with disabilities were particularly at risk of experiencing discrimination when seeking maternal health care and services, although men with disabilities were also affected.

“When it comes to the time of birth, they [midwives] say ‘Have you seen? You! You climb on the bed’. You cannot see where the bed is. You need to be directed [to where it is]. ‘You climb. Do it, as you were doing it when you were getting the child!’” *(Woman, vision impairment, Gulu)*

“When they [deaf women] are pregnant, it’s very hard to receive antenatal services and care. When they go to the hospital, on some occasions, they end up having a caesarian because there is a gap in communication between the person and the health service provider.” *(Woman, hearing impairment, Gulu)*

“You know there are some people who are disabled, they just crawl and they’re unable to get from their places to the hospital. When they’re screened and they get that they’re HIV positive, they are supposed to come here and get medicine on a routine basis, but what they do is to send those who are able with their medical forms to come and get for them their medicine.” *(Man, physical impairment, Omoro)*

The non-participant observations (Table 3) corroborated what most participants shared in terms of inaccessibility of services, especially for health centres located further away from Gulu town, the major peri-urban area in Northern Uganda. Most health facilities were largely not physically accessible, combined with the absence of adapted toilets and maternity beds. Further, none of the observed health facilities provided sign language interpretation. At the structural level, the Ministry of Health requires all health facilities to maintain a patient registry, which includes a specific column to collect disability data. However, this column was often left empty or was irregularly filled out by health providers, therefore not identifying disabled people who sought treatment. Table 3 summarises the main observations of the seven health facilities visited.

**Table 3.** Findings of non-participant observations

	Private-not-for-profit referral hospital	Public referral hospital	Private-not-for-profit health centre level III	Public health centre level III
Availability of accessible ramps and in acceptable condition	Yes	Yes	No	No
Availability of accessible toilets or separate toilets for people with disabilities	No	No	No	No
Availability of accessible maternity beds	No	N/A	No	No
Availability of accessible signage or sign language interpretation	No	No	No	No
Availability of a disability desk	No	No	No	No
Regular completion of Column 16 on Disability in the Ministry of Health’s Patient Registry	No	N/A	No	No

### Expectations to exercise rights despite implementation challenges

Despite diverse levels of knowledge about specific disability-related legislation and policy in relation to SRH, participants knew about the existence of policy implementation challenges. These included a lack of policy enforcement, limited budget allocation for disability issues, limited skills among health providers to provide adapted services, lack of accessible mass education and weaknesses among elected bodies, including disabled officials, to promote and protect the rights of people with disabilities. These policy implementation gaps had a direct impact on their experiences when using SRH services. People with disabilities clearly expressed that they expected to be able to exercise their rights, despite having a vague sense of what the pro-disability policies actually entailed. Most participants we interviewed expressed the idea of having the right to establish intimate relationships, become parents, use health services, work, study and simply be, despite powerful societal pressures to fit in and be “normal” (Woman, physical impairment, Amuru; Man, hearing impairment, Gulu).

“I know that the rights of persons with disabilities are equal with others. What a normal person can do, a disabled person can do … someone with disability has the right to produce [have children], has the right to study, has the right to work, like any other person.” *(Woman, physical impairment, Amuru)*

### Importance of policy implementation

In the view of many participants, the extension and translation of legislation and policy implementation would enable people with disabilities to use SRH services in which health providers are culturally competent and provide high quality, respectful, and dignified care to people with disabilities. For others, policy implementation is operationalised through specific policy translation and accessibility measures, such as the provision of adapted maternity beds and ramps, necessary for them to access services. Without these facilitating factors, a gap is created between policy adoption and SRH service utilisation.

“When you are pregnant, the laws [should] always take care of you when you come to the hospital, those nurses, those doctors, the laws always say they should give enough services without failure, without ignoring any person at all. This is what I know.” *(Man, vision impairment, Amuru)*

“The thing is that these policies are just on paper! … When it comes to important documents like the Disability Act or the CRPD, nobody knows about it. People don’t read, those laws are not promoted in the communities. They are in the hands of only those politicians and strong men, and strong organisations in Kampala. But ordinary people don’t understand. Our leaders are a problem, but the policies are there. I have copies with me here. Am not a legislator, I cannot fight alone (laugh), you see. That’s the problem. When you go to the health centre, it will be you alone, telling the nurse to do thing like this, construct a ramp there … They will just look at you. Our challenge is implementation.” *(Man, hearing impairment, Gulu)*

### Multiple concrete solutions and recommendations proposed

To improve their sexual health and reduce experiences of discrimination regarding SRH services, people with disabilities expressed a range of recommendations. They went beyond identifying problems of policy implementation in the context of SRH service utilisation and clearly cited multi-level solutions that are motivated by social justice and equity which have the potential to improve the lives of people with disabilities. At the micro level, participants proposed that people with disabilities be empowered through education opportunities and community participation in awareness-raising activities. A woman with a physical impairment, from Amuru, recommended the following: “*They should teach people with disabilities, because there are some that fear even to get pregnant. So, they should teach [people with] disabilities”.* Another study participant suggested more social participation.

“The persons [people with disabilities] in the village should participate, they need to first understand these legislation and laws. And they themselves would see if it is truly being followed through. Then, they can start playing an active role in pushing for such services and advocating for such services.” *(Man, hearing impairment, Gulu)*

At the meso level, they insisted that family members and service providers be trained on the diversity of experiences of people with disabilities and on SRH rights, coupled with better accessibility of basic infrastructure (e.g. toilets and ramps) as well as information and services (e.g. provision of sign language interpretation). Specifically, a woman with vision impairment, from Gulu, recommended that “*The family needs to be educated on how people with disabilities can be treated, so they are also able to help themselves”.* Improvement in making communication more accessible was also recommended:

“All these health service providers should learn sign language. It will be easy for anyone who is deaf to access services. For example, a pregnant woman would easily communicate to any person in maternity. She can be helped when a doctor knows simple signs.” *(Man, hearing impairment, Omoro)*

At the macro level, people with disabilities highlighted the necessity to move beyond a policy on “paper” toward the implementation of measures that will have a positive impact on the sexual rights of people with disabilities, such as allocating adequate budgets for the expansion and development of disability-sensitive services. Indeed, participants indicated that the lack of data on people with disabilities was a social justice challenge and recommended that more research be conducted to document and collect information on disabilities, such as impairment type.

“For example, at the sub-county level, they don’t have the capacity of having transport to move deep down in the village there. But if you go at the sub-county to check on their budget, they don’t have a budget for that. That is if the government can put some budget, it would help them move to villages, to the grass root, to persons with disabilities.” *(Man, vision impairment, Amuru)*

“I feel another thing is … to do research. A recommendation. One research about persons with disabilities and the differences [how] to help service providers and law makers to be able to understand how best to serve persons with disabilities, without just putting a law or a policy without doing a proper research to understand disability itself in relation to reproductive health service provision. A person who has experience in the difference in disabilities would serve people with disabilities the best way.” *(Man, vision impairment, Amuru)*

## Discussion

This paper provides a novel contribution to the literature by examining how people with disabilities perceived their utilisation of SRH services in the context of legislation and policy implementation in Northern Uganda. We report three major findings. First, through an intersectionality-informed analysis, we were able to broaden the evidence base regarding the complexities of experience across the diversity of women and men with disabilities. People with disabilities, women in particular, experienced multiple concurrent intersections related to gender, disability, and violence when using various SRH services. These intersections were complex and multilayered, with disability interconnected with both gender and violence. It has been reported in the literature that the prevalence of all forms of violence, including sexual violence, is higher among people with disabilities relative to people without disabilities.^10^ A systematic review and meta-analysis conducted among adults with disabilities in sub-Saharan Africa found that people with disabilities were more at risk for HIV compared to non-disabled people, with an increasing gradient of risk for HIV based on gender and disability.^9^ The reasons cited for this heightened vulnerability to HIV were limited access to HIV prevention and a higher risk of sexual violence.^35^ Our study also complements the findings of a meta-synthesis on gender, disability, and reproductive health in sub-Saharan Africa which reported the exacerbation of gendered roles among women with disabilities who sought reproductive health services.^36^ People with disabilities, especially women, were considered “not normal” and were expected not to have children. The societal norm for “normalcy” conferred to abled-bodies highlighted the denigration of and the insidious impact of ableism, upon disabled bodies.^37^ According to intersectionality theory, power structures such as ableism shape the experiences of privileges for “able-bodied” and penalties to those who are disabled.^25^

Second, women and men with disabilities experienced a wide range of attitudinal, communication, and physical barriers when accessing and using SRH services, irrespective of the type of health facilities being public or private-not-for-profit. While the finding related to barriers faced by people with disabilities is not new and supports what has already been reported in the literature,^7,8,38^ the finding related to the similarity of the challenges faced by people with disabilities across health facility type is novel. Past studies have described higher levels of satisfaction, a proxy to quality of care, among a wide range of users of faith-based (private-not-for-profit) health providers, compared to public facilities in Africa.^34^ Across the continent, faith-based health providers and organisations are seen as playing a key role in service provision in weakened health systems, such as in post-conflict settings.^34^ People with disabilities were not passive when discussing the discriminatory barriers to the use of SRH services. They insisted on their sexual rights in addition to their reproductive health rights, and that these rights be treated as equal to those of non-disabled people. This is in sharp contrast to the local social silence surrounding disability and sexuality.^2^

Third, given the opportunity to express what they thought they knew about existing laws and policies promoting their rights, people with disabilities were consciously reflecting on their self-awareness of the relationships between policy and SRH service utilisation.^25^ This analysis also supports moving beyond individual risk factors and highlights the need to examine power structures, such as ableism, which gives unearned privileges to abled-bodies while oppressing people with diverse bodies and abilities.^39,40^ As per the recommendations made by people with disabilities, a transformative shift is required in how society views and considers people with disabilities when insisting on their disability and SRH rights.^25^ The people with disabilities explicitly suggested means of being better empowered at the community level as well as implementable and enforceable actions in the health system and at a national level, and positioned themselves as active policy actors. This shows a desire and commitment to social justice and equity for people with disabilities within a larger system of socio-political structures^23^ and is coherent with the transformative nature which the intersectional approach is promoting.^25^

### Limitations

The perspectives of other policy actors – namely health service providers, disabled people’s organisations, international organisations, and national policy-makers interviewed in the study – were not included in this paper. Our goal was to present an in-depth analysis from the perspective of individuals with disabilities and we prioritised their voices as they are often ignored and silenced.^2^ As a result, the perspectives of other policy actors are absent from this analysis, which is therefore not addressing any possible convergent or divergent findings at the micro level. Nonetheless, the rich accounts from the study participants provided critical insight into their experiences and constitute the foundation for further differential analyses.^41^ We did not include the fully privatised health facilities among cases to contrast. Having this third group of health facilities could have provided a different understanding of SRH service utilisation. However, given that they are less numerous than private-not-for-profit and public health facilities in the three target districts,^42^ and that their services are generally costly, it is less likely that people with disabilities would use their services.^34^ Finally, we used translation and sign language interpretation during interviews and focus groups, and cross-cultural translation and interpretation may have added another level of meaning.^43^ To mitigate this risk, we piloted our interview tools, developed a bilingual glossary of key research and SRH vocabulary, followed by verifying the translations.

### Conclusion and implications for policy and programmes

This study provided substantial evidence of the intersecting discrimination experienced by women and men with disabilities and the numerous barriers they face using SRH services. An intersectionality-informed analysis highlighted the complex relationships and interactions between gender, disability, the utilisation of SRH services, and the expectation that people with disabilities can exercise their rights despite policy implementation hurdles. The concrete multiple level recommendations put forth by people with disabilities are already enshrined in the disability rights articulated in the CRPD, as ratified by Uganda in 2008. At the macro level, the findings presented here provide evidence-based arguments to the current national review process of the Ugandan Disability Act to ensure that both policy and its implementation align with the objectives, scope, and language promoted in the CRPD. People with disabilities recommended tighter enforcement of policy implementation through improved budget allocation for disability and more accountability from policy-makers and implementers. At the meso level, people with disabilities insisted that health professionals as well as family members be sensitised and trained on disability-sensitive SRHR to remove attitudinal, physical and structural barriers. The experiences and recommendations of people with disabilities should be used to inform the monthly and annual review meetings of District Health and Community Development Offices for further monitoring and follow-up. Within specific health facilities, recommendations pertaining to accessibility improvements can be integrated during two specific periods: 1) during annual strategic review and planning meetings, and 2) during decision-making processes for service and technical resource budget allocation. At the micro level, people with disabilities further stressed the importance of being empowered through social participation, education and sensitisation on their SRHR. In conclusion, capitalising on the global objectives for universal healthcare access, “leaving no one behind” particularly matters for women and men with disabilities when seeking SRH services. Universal health coverage can be operationalised in actionable measures, where its universality meets with social justice.^44^

## Supplementary Material

Supplementary Files 1-4
