# Supplementary material for: The intersectional jeopardy of disability, gender and sexual and reproductive health: experiences and recommendations of women and men with disabilities in Northern Uganda
Source: Sex Reprod Health Matters. 2020 Jun 19;28(2):1772654. doi: 10.1080/26410397.2020.1772654 (PMC7887920; doi:10.1080/26410397.2020.1772654)
Supplement: Supplementary Files 1-4 [file ZRHM_A_1772654_SM0608.docx]

**Supplementary file 1: Phases of the field research**

| **Phase 1: Phase-in** | - Settling down in a temporary accommodation - Introduction to the local stakeholders and exploration of the Lacor Hospital compound - Identification of and exchanges with key knowledge brokers (n=30) - Recruitment interview process of potential Research Assistant (RA) candidates (n=11) - Research team recruitment:   - Orientation of two RAs: one woman and one man, with experience in qualitative data collection and working with people with disabilities   - Identification of reliable *boda-boda* (moto-taxi) drivers (n=2) to drive to villages   - Identification of qualified local sign language interpreters (n=3) - Elaboration with RAs of a glossary of key research and SRH vocabulary in English and Luo/Acholi - Field testing of the interview and focus group guidelines for people with different types of impairments - Start and maintenance of a methodological and reflexivity logbook |
| --- | --- |
| **Phase 2:**  **Data collection** | - Courtesy visits to all District Commissioners, Medical Officers (DHO) and Community Development Officers (DCDO), and obtention of official permissions to collect data in target districts - Meeting with local disabled people’s organisations (DPOs) and organisations working for people and children with disabilities (n=6) - Community mobilisation by Village Health Teams (VHT) of health facilities and volunteers with disabilities - In-depth semi-structured interviews of people with disabilities (n=32) and focus groups (n=2) - 50.2 hours of recording and 956 pages of transcription completed - Preliminary visits and non-participant observations of health facilities (n=7) |
| **Phase 3: Phase-out** | - Courtesy follow-up visits to all DHO, DCDO and Commissioners to provide feedback and present preliminary findings - Organisation of preliminary finding presentations (n=5), including to DPOs - Organisation of a 2-day orientation training on disability-friendly health service provision to more than 30 health staff and managers of health facilities (n=6). Co-facilitators were four people with disabilities (2F and 2M) and two sign language trainers (one of whom is deaf) - Farewell to partners and stakeholders, and host community at the Lacor Hospital |

**Supplementary file 2: COREQ Checklist for Qualitative Research**

| **Domain 1: Research team and reflexivity** | |
| --- | --- |
| *Personal characteristics* | |
| 1. Interviewer/facilitator | Yes |
| 1. Credentials (of researchers) | Yes |
| 1. Occupation (of researchers at the time of the study) | Yes |
| 1. Gender | Yes |
| 1. Experience and training | Yes |
| *Relationship with participants* | |
| 1. Relationship established | Yes |
| 1. Participant knowledge of the interviewer | Yes |
| 1. Interviewer characteristics | Yes |
| **Domain 2: Study design** | |
| *Theoretical framework* | |
| 1. Methodological orientation and theory | Yes |
| *Participant selection* |  |
| *Sampling* | |
| 1. Method of approach | Yes |
| 1. Sample size | Yes |
| 1. Non-participation (from selected sample) | None |
| 1. Setting of data collection | Yes |
| 1. Presence of non-participants | No |
| 1. Description of sample | Yes |
| *Data collection* | |
| 1. Interview guide | Yes |
| 1. Repeat interviews | No |
| 1. Audio/visual recording | Yes |
| 1. Field notes | Yes |
| 1. Duration | Yes |
| 1. Data saturation | Yes |
| 1. Transcripts returned | No |
| **Domain 3: Analysis and findings** | |
| *Data analysis* | |
| 1. Number of data coders | Yes |
| 1. Description of the coding tree | Available upon request |
| 1. Derivation of themes | Yes |
| 1. Software used | Yes |
| 1. Participant checking | Yes |
| *Reporting* | |
| 1. Quotations presented | Yes |
| 1. Data and findings consistent | Yes |
| 1. Clarity of major themes | Yes |
| 1. Clarity of minor themes | Yes |

## **Supplementary file 3: Semi-structured interview guide with people with disabilities**

| **Date:** | Location: |
| --- | --- |
| Interviewer(s): | **Presence of a local sign language interpreter**?  Y / N |
| Interviewee’s title and/or organisation: | Membership of interviewee to a disabled people’s organisation?  Y / N |
| Sex of interviewee:  F / M  **Age:** | **Which disability the interviewee identify her/himself to?**   - Physical - Vision - Hearing - Communication - Concentration and remembering - Multiple |

**Introduction**

Thank you for accepting to be talk to us today. This interview will take approximately one hour of your time. We can take breaks in between if needed, please let us know. The information that you will provide will be kept confidential. Your answers will not be associated with your name, unless you want to be identified. Your answers will only be shared with other research team members and will be compiled with other answers from various participants with disabilities.

I am going to ask you questions about your perceptions (i.e. ideas, point of views) on how laws, health policies are related to the use of sexual and reproductive health services by women and men with disabilities, in particular in the post-conflict Northern region of Uganda. At any time, you may ask for clarification if questions are not clear to you.

### **Anonymity**

Despite being recorded, I would like to assure you that the discussion will be anonymous. The tapes will be kept safely in a locked facility until they are transcribed word for word, then they will be destroyed. The transcribed notes of the interview will contain no information that would allow individual subjects to be linked to specific statements. You should try to answer and comment as accurately and truthfully as possible. If there are any questions or discussions that you do not wish to answer or participate in, you do not have to do so; however please try to answer and be as involved as possible.

##### **Guiding questions**

1. **Descriptive questions in relation to the IBPA**

- Tell us a bit more about yourself, age, marital status, disability, education, employment, etc.?
- What do you know about sexual and reproductive health services available in Uganda?
- Which ones are you using?
- What kinds of challenges are you facing as a person with disabilities, or other people with disabilities, are facing in the utilisation of these sexual and reproductive health services?
- Are there any differences in the utilisation of these services with other groups of people in Uganda, for example those who are not disabled and/or who are living in other regions that were not so affected by the last armed conflict?
- How do you think the use of sexual and reproductive health services can be influenced by Ugandan legislation and health policy?
- Are you aware of any legislation and health policy that are supposed to protect and promote the rights of people with disabilities in Uganda?
- If yes, which ones?
- If not, why?
- So if we look back at the question of this study, what could be the linkages or the relationships between legislation, health policy and sexual and reproductive health services by people with disabilities, in the post-conflict Northern region of Uganda?

1. **Transformative questions in relation to the IBPA**

- How can we improve the access to and utilisation of sexual and reproductive health services by people with disabilities in Uganda and especially in the Northern post-conflict region?
- What are the roles of people with disabilities - service providers - local NGOs – international organisations – national policy-makers, in promoting transformative action for improved access to and utilisation of SRH services by people with disabilities?

##### **Follow-up during focus groups**

Before we depart from one another, if needed, would you be interested to be part of a focus group with other people with disabilities to deepen our understanding of what we just talked about?

Y / N

##### **Conclusion**

- Thank you for participating. This has been a very successful discussion
- Your opinions will be a valuable asset to the study
- We hope you have found the discussion interesting
- I would like to remind you that any comments featuring in this report will be anonymous
- If you would like to review some of the accuracy of your statements when we will write the report, please let me know if you would like to be contacted. If yes: email or local phone number

## **Supplementary File 4: Focus group interview guide with people with disabilities**

| **Date:** | | | Location: | |
| --- | --- | --- | --- | --- |
| Interviewer(s): | | | Presence of local sign language interpreter:  Y / N | |
| Interviewee’s title and/or organisation: | **Sex of participants:** | **Age:** | Disability type:   - Physical - Vision - Hearing - Communication - Concentration and remembering - Multiple | **Membership to any disabled people’s organisations:**  Y /N  **Which one?** |
| **Notes (e.g. if anyone came accompanied, etc.):** | | | | |

**Welcome** and introduction of researcher/research assistant (and local sign language interpreter, if needed).

**Overview**

We have met during individual interviews. Today, we are meeting as you have accepted to be interviewed in a group. Again, the objective of this study is to look at the relationships between laws, health policies and utilisation of sexual and reproductive health services by people with disabilities in the post-conflict Northern region of Uganda.

**Anonymity**

Despite being taped, I would like to assure you that the discussion will be anonymous. The tapes will be kept safely in a locked facility until they are transcribed word for word, then they will be destroyed. The transcribed notes of the focus group will contain no information that would allow individual subjects to be linked to specific statements. You should try to answer and comment as accurately and truthfully as possible. I and the other focus group participants would appreciate it if you would refrain from discussing the comments of other group members outside the focus group. If there are any questions or discussions that you do not wish to answer or participate in, you do not have to do so; however please try to answer and be as involved as possible.

##### **Warm-up**

First, I would like everyone to introduce themselves. Can you tell us your name and where do you come from?

##### **Guiding questions**

- What are the types of sexual and reproductive health services you have tried to use or have used?
- Where did you go for these services?
  - Probe: type of health facility, i.e. private not-for-profit-based or government-based?
- What are the facilitating factors and barriers in accessing and utilising sexual and reproductive health services in the Northern region and why?
- Have you, or anyone you know, been treated differently because of age, sex, disability, place of residence, etc.?
  - Probe: Can you explain?
- Have these treatments been always the same/different, for example since the adoption of disability laws and policies in Uganda?
  - Probe: Have you seen any changes in time, for example, since 2006, the year of the adoption of the Disability Act in Uganda?
- How do you think can these facilitating factors be enhanced and these barriers be handled by yourselves as people with disabilities, health facilities (which type?), service providers, community leaders and national policy-makers?

##### **Conclusion**

- Thank you for participating. This has been a very successful discussion
- Your opinions will be a valuable asset to the study
- We hope you have found the discussion interesting
- I would like to remind you that any comments featuring in this report will be anonymous
- If you would like to review some of the accuracy of your statements when we will write the report, please let me know if you would like to be contacted. If yes: email or local phone number
